# Cargo Delivery into the Brain by *in vivo* identified Transport Peptides

**DOI:** 10.1038/srep14104

**Published:** 2015-09-28

**Authors:** Eduard Urich, Roland Schmucki, Nadine Ruderisch, Eric Kitas, Ulrich Certa, Helmut Jacobsen, Christophe Schweitzer, Alessandra Bergadano, Martin Ebeling, Hansruedi Loetscher, Per-Ola Freskgård

**Affiliations:** 1 Neuroscience Discovery and Translation Area, Pharma Research and Early Development (pRED), Roche Innovation Center Basel, F. Hoffmann-La Roche Basel, Switzerland; 2 Pharmaceutical Sciences, Pharma Research and Early Development (pRED), Roche Innovation Center Basel, F. Hoffmann-La Roche Basel, Switzerland; 3 Medicinal Chemistry, Pharma Research and Early Development (pRED), Roche Innovation Center Basel, F. Hoffmann-La Roche Basel, Switzerland

## Abstract

The blood-brain barrier and the blood-cerebrospinal fluid barrier prevent access of biotherapeutics to their targets in the central nervous system and therefore prohibit the effective treatment of neurological disorders. In an attempt to discover novel brain transport vectors *in vivo*, we injected a T7 phage peptide library and continuously collected blood and cerebrospinal fluid (CSF) using a cisterna magna cannulated conscious rat model. Specific phage clones were highly enriched in the CSF after four rounds of selection. Validation of individual peptide candidates showed CSF enrichments of greater than 1000-fold. The biological activity of peptide-mediated delivery to the brain was confirmed using a BACE1 peptide inhibitor linked to an identified novel transport peptide which led to a 40% reduction of Amyloid-β in CSF. These results indicate that the peptides identified by the *in vivo* phage selection approach could be useful transporters for systemically administrated large molecules into the brain with therapeutic benefits.

Central nervous system (CNS) targeted therapeutic research is mostly directed towards identification of optimized drugs and formulations demonstrating properties for CNS exposure whilst less effort is given to the discovery of mechanisms allowing active delivery of drugs to the brain. This is now beginning to change as drug delivery, especially for large molecules, is an integral part of modern neuroscience drug development. The CNS environment is highly protected by the brain vascular barrier system consisting of the blood-brain barrier (BBB) and the blood-cerebrospinal fluid barrier (BCSFB)^1^ which make delivery of drugs to the brain such a challenging task^1^,^2^. It has been estimated that almost all large molecules and more than 98% of small molecular weight drugs are excluded from the brain^3^. This is why identification of novel brain transport systems, enabling efficient and specific delivery of therapeutics to the CNS is of high importance^4^,^5^. The BBB and BCSFB nevertheless, also provide a great opportunity for drug delivery, as it penetrates and infiltrates the whole brain structure with its extensive vascular network. Consequently, current efforts using non-invasive brain delivery approaches are primarily based on receptor-mediated transport (RMT) mechanisms using endogenous receptors at the BBB^6^. Although key progress has recently been made using the Transferrin Receptor pathway^7^,^8^, novel transport systems with improved properties are needed for further refinements. To this end, our goal is to identify peptides capable of mediating transport into the CSF as they in principle could be applied to deliver large molecules into the CNS or for the discovery of novel receptor pathways. In particular, specific receptors and transporters of the brain vasculature (BBB and BSCFB) could serve as potential targets for the active and specific delivery of biotherapeutics. The cerebrospinal fluid (CSF), a secretory product of the choroid plexus (CP) is in direct contact with the brain interstitial fluid through the subarachnoid and ventricular space^4^. It has recently been shown that there is an excessive influx of subarachnoid CSF into the brain interstitium^9^. We want to access the brain parenchymal space by exploiting this subarachnoid influx route or by direct crossing of the BBB. To achieve this goal, we have implemented a robust *in vivo* phage selection strategy that would ideally identify peptides that traffic either of these two distinct pathways.

We now describe a continuous *in vivo* CSF sampling phage display screening approach combined with high throughput sequencing (HTS) to monitor the initial selection rounds where library diversity is highest. The screening was performed in conscious rats permanently implanted with *cisterna magna* (CM) cannula to avoid blood contamination. Importantly, this approach selects for both brain targeting and also for peptides having transport activities at the brain vascular barriers. We have used T7 phage because of their small size (~60 nm)^10^ and hypothesized that they fit into transport vesicles allowing transcellular crossing over the endothelial and/or epithelial cellular brain barriers. After four rounds of panning, phage populations were isolated that displayed strong *in vivo* CSF enrichment and brain microvascular association. Importantly, we were able to validate our findings by demonstrating that prioritized and chemically synthesized top candidate peptides were able to transport a protein cargo into the CSF. Firstly, a pharmacodynamic CNS effect was established by combining a lead transport peptide with a BACE1 peptide inhibitor. Besides demonstrating that the *in vivo* functional screening strategy can identify novel brain transporter peptides that are competent carriers of protein cargos we anticipate that similar functional selection methods will also become important for identification of novel brain transport pathways.

## Results

### T7 phage peptide library design and profiling

A random 12-mer linear T7 phage peptide library was designed and produced with a diversity of approximately 10^9^ based on plaque forming units (pfu) after the phage packing step (see Material and Methods). Importantly, we carefully profiled this library before *in vivo* panning. A sample of the stock phage library was subjected to PCR amplification using modified primers generating amplicons directly applicable to HTS (Supplementary Fig. 1a). Due to a) sequencing errors from the HTS procedure^11^, b) the influence from the primers (NNK)_1-12_ quality; and c) the occurrence of “wild-type” (wt) phages (backbone insert) in the stock library, a sequence filtering procedure was implemented to extract only validated sequence information (Supplementary Fig. 1b). These filtering steps were applied for all HTS sequenced libraries. For the stock library, a total of 233′868 reads was obtained of which 39% passed the filtering criteria and used to profile the library and subsequent rounds of selections (Supplementary Fig. 1c-e). The lengths of the reads were predominantly multiples of 3 base pairs with a peak at 36 nucleotides (Supplementary Fig. 1c) confirming the (NNK)_1-12_ library design. Notably, approximately 11% of the stock library members contain the “wild-type” (wt) backbone 12-mer insert PAGISRELVDKL and almost half of the sequences (49%) contained either insertions or deletions. HTS of the stock library confirmed that the peptide diversity in the library was high as more than 81% of the peptide sequences were found only once and only 1.5% appeared in ≥4 copies (Supplementary Fig. 2a). The frequency of amino acid (aa) in the stock library at all 12 positions showed a good correlation with that expected from the numbers of codons generated by the NKK degenerated library (Supplementary Fig. 2b). The observed frequencies of aa residues coded by these inserts correlated well (r = 0.893) with the calculated frequencies (Supplementary Fig. 2c). Preparation of phage libraries for injection included an amplification and endotoxin removal steps. This has previously been shown to potentially decrease and bias the diversity of phage libraries^12^,^13^. We therefore sequenced and compared a plate amplified phage library subjected to an endotoxin removal step with the stock library to assess aa frequencies. There was a strong correlation (r = 0.995) between the stock library and the amplified and purified library (Supplementary Fig. 2d) suggesting that there was no major bias resulting from competition amongst clones using plate amplification of T7 phages. This comparison was based on the frequency of tripeptide motifs in each library as a complete coverage of the library diversity (~10^9^) was not possible, even with HTS. The analysis of the aa frequency at each position showed a slight position-dependent bias at the last three positions in the injected library (Supplementary Fig. 2e). In summary, we conclude that the quality and diversity of the library was acceptable and that only minor changes in the diversity were observed because of the amplification and preparation of the phage library between rounds of selections.

### *In vivo* selection of CSF-homing phage peptides

**Figure 1:**
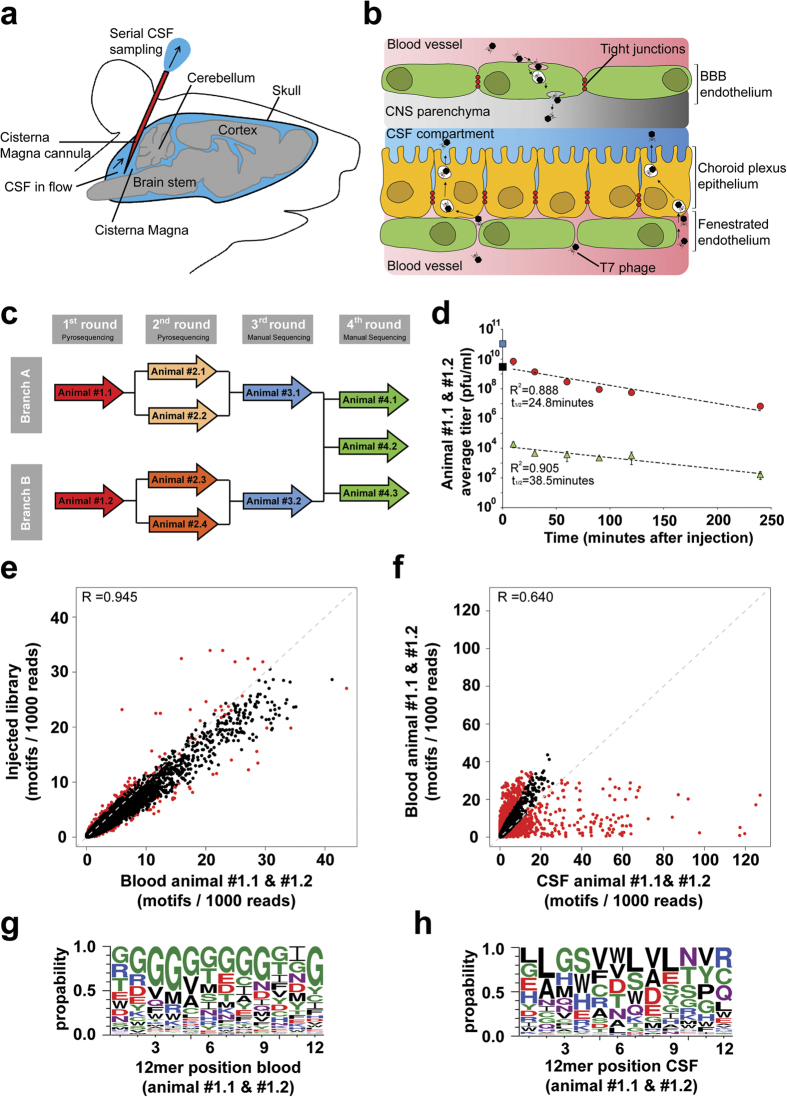
Functional *in vivo* phage-display screening for brain penetrating peptides. (**a**) The methodological setup used for repeated cerebro spinal fluid (CSF) sampling from the cisterna magna. (**b**) Diagram showing cellular location of central nervous system (CNS) barriers and selection strategy which was used to identify peptides that cross the Blood Brain Barrier (BBB) and Blood CSF Barrier. (**c**) Flow chart description of the *in vivo* phage display screening. In every selection round phages are i.v. injected (animal ID inside arrows). Two independent selection branches (**A,B**) were kept separately until the 4^th^ selection round. For the 3^rd^ and 4^th^ selection rounds every CSF recovered phage clone was manually sequenced. (**d**) Kinetics of phages recovered from blood (red circles) and CSF (green triangles) during the 1^st^ selection round of two cannulated rats after i.v. injection of the T7 peptide library (2 × 10^12^ phages/animal). Blue square represents the averaged initial blood phage concentrations calculated from the injected phage amount considering the total blood volume. The black square displays the y-axis intercept of the straight line extrapolated from the blood phage concentrations. (**e,f**) Relative frequencies and distribution of the motifs representing all possible overlapping tripeptides found within the peptides. Displayed are the numbers of motifs found within 1000 reads. Motifs which are significantly (p < 0.001) enriched are highlighted by red colored dots. (**e**) Correlation scatter plots comparing the relative tripeptide motif frequencies of the injected library vs. the blood derived phages of animals #1.1 and #1.2. (**f**) Correlation scatter plots comparing the relative tripeptide motif frequencies of the blood and CSF recovered phages of animals #1.1 and #1.2. (**g,h**) Sequence logo representation of phages enriched in the blood **(g)** compared to the injected library and enriched in the CSF **(h)** compared to blood after one round of *in vivo* selection in both animals. The size of the single letter code represents the frequency of occurrence of that amino acid at a given position. Green = polar, purple = neutral, blue = basic, red = acidic & black = hydrophobic amino acids. Figure 1a,b was designed and produced by Eduard Urich.

A surgically implanted cannula into the CM of conscious rats enabled continuous CSF sampling to facilitate the identification of intravenously (i.v.) injected T7 phages that cross the BBB and/or BCSFB (Fig. 1a–b). We used two independent selection branches (branches A and B) over the first three *in vivo* selection rounds (Fig. 1c). We successively increased the selection stringency by reducing the total amount of injected phages in the first three selection rounds. For the fourth round of panning we pooled the samples from branch A and B and performed three additional independent selections. To investigate the *in vivo* properties of the T7 phage particle in this model, wt phages (backbone PAGISRELVDKL insert) were injected into the tail vein of a rat. Recovery of phages from the CSF and blood at different time points showed that the relatively small icosahedral T7 phages have a rapid phase of initial clearance from the blood compartment (Supplementary Fig. 3). Based on the injected titer and the rat blood volume we calculated that only about 1% of the injected dose of wt phage was detected in the blood 10 min post i.v. injection. After this initial and rapid drop a slower first-order clearance rate with a half-life of 27.7 min was measured. Importantly, only very few phages were recovered from the CSF compartment demonstrating a low background of migration of wt phage into the CSF compartment (Supplementary Fig. 3). On average only about 1 × 10^−3^% of the blood T7 phage titer and 4 × 10^−8^% of the initially injected phage could be detected in the CSF over the entire sampling period (0–250 minutes). Notably, the half-life of wt phages in CSF (25.7 min) was similar to what was observed in blood. These data suggest that the barriers separating the CSF compartment from the blood are intact in the CM cannulated rats, and thus allow the *in vivo* selection of phage libraries for the identification of clones that are prone to be transported from the blood into the CSF compartment.

**Figure 2:**
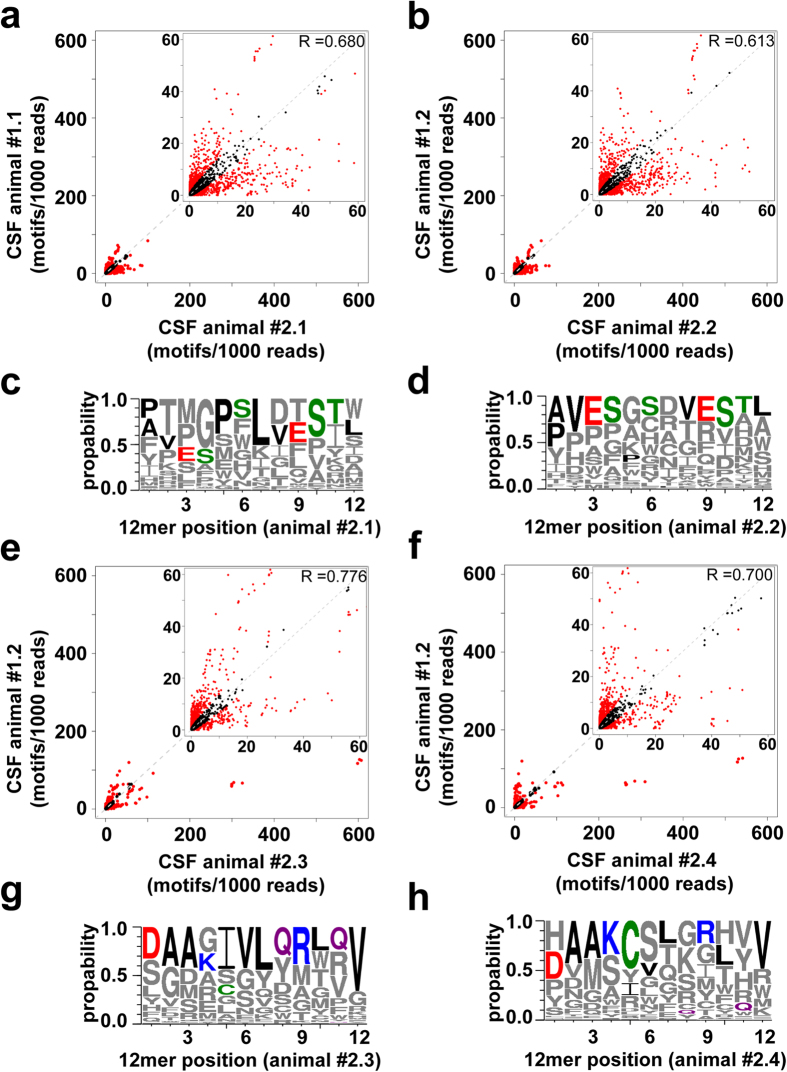
Motifs and peptides enrichments in the CSF over two consecutive rounds of functional *in vivo* phage-display selection. All CSF recovered phages from the 1^st^ round of each animal (animals #1.1 & #1.2) were pooled, amplified, HT sequenced and re-injected together (2 × 10^10^ phages/animal) into 2 CM cannulated rats each (#1.1 → #2.1 & #2.2, #1.2 → #2.3 & #2.4). (**a,b,e,f**) Correlation scatter plots comparing the relative tripeptide motif frequencies of all CSF recovered phages in the 1^st^ versus the 2^nd^ selection round. Relative frequencies and distribution of the motifs representing all possible overlapping tripeptides found within the peptides in both directions. Displayed are the numbers of motifs found within 1000 reads. Motifs which are significantly (p < 0.001) selected or de-selected in one of the compared libraries are highlighted by red colored dots. (**c,d,g,h**) Sequence logo representation based on all 12 amino acid long sequences enriched in the CSF in the 2^nd^ versus the 1^st^ round of the *in vivo* selection. The size of the single letter code represents the frequency of occurrence of that amino acid at a given position. For the logo representation the frequency of the CSF recovered sequences from individual animals between the two selection rounds were compared and the enriched sequences in the 2^nd^ round are displayed: (***c***) #1.1–#2.1 **(d**) #1.1–#2.2 **(g)** #1.2–#2.3 and **(h)** #1.2–#2.4. Most enriched amino acids at a given position in (**c,d**) animals #2.1 and #2.2 or in (**g,h**) animals #2.3 and #2.4 are displayed in colors. Green = polar, purple = neutral, blue = basic, red = acidic & black = hydrophobic amino acids.

We injected the phage peptide library into two CM instrumented rats (branch A and B) and recovered phages from the CSF and blood (Fig. 1d). The initial rapid clearance was much less pronounced for the library as compared to the wt phage. The average half-life in blood of the injected library for the two animals was 24.8 min which is similar to the wt phage, while the half-life in CSF was 38.5 min. The Blood and CSF phage samples from each animal were subjected to HTS and all identified peptides were analyzed for the presence of short tripeptide motifs. Tripeptide motifs were chosen as they provide the minimal framework for structural formation and peptide-protein interaction^14^,^15^. We found a good correlation of the motif distribution between the injected phage library and the recovered clones in the blood of both animals (Fig. 1e). The data indicates that the composition of the library is only slightly enriched in the blood compartment. Amino acid frequency and consensus sequences were further analyzed at each position by adapting the web logo software^16^. Interestingly, we detected a strong enrichment of glycine residues in the blood (Fig. 1g). By comparing the blood with the CSF sampled clones a strong selection and some de-selection of motifs was observed (Fig. 1f) and certain amino acids occur preferentially at a given position within the 12-mer (Fig. 1h). Notably, there was a striking difference for the individual animals in the CSF while the glycine enrichment in blood was seen in both animals (Supplementary Fig. 4a-j). After the stringent filtering of sequence data, a total of 964 and 420 unique 12-mer size peptides were obtained in the CSF of animal #1.1 and #1.2, respectively (Supplementary Fig. 1d-e). The isolated phage clones were amplified and subjected to a second round of *in vivo* selection. The recovered phages from the second selection round for each individual animal were subjected to HTS and all identified peptides were used as input data for the motif recognition software to analyze the occurrence of tripeptide motifs (Fig. 2a,b,e–f). We observed a further selection and de-selection of numerous motifs in the CSF within the selection branches A and B compared to the CSF recovered phages of the first round (Fig. 2). The web logo algorithm was applied to determine if they represent different patterns of consensus sequences. Clear similarities between the CSF recovered 12-mer sequences within the selection branch A (Fig. 2c,d) and branch B (Fig. 2g,h) were observed. The pooled analysis within each branch shows the different selection profile for a 12-mer peptide (Supplementary Fig. 5c,d) and the increase CSF/blood titer ratio for the pooled clones over time after the second round of selection as compared to the first round (Supplementary Fig. 5e).

### *In vivo* phage selection identifies CSF enriched phage displayed peptides

**Figure 3:**
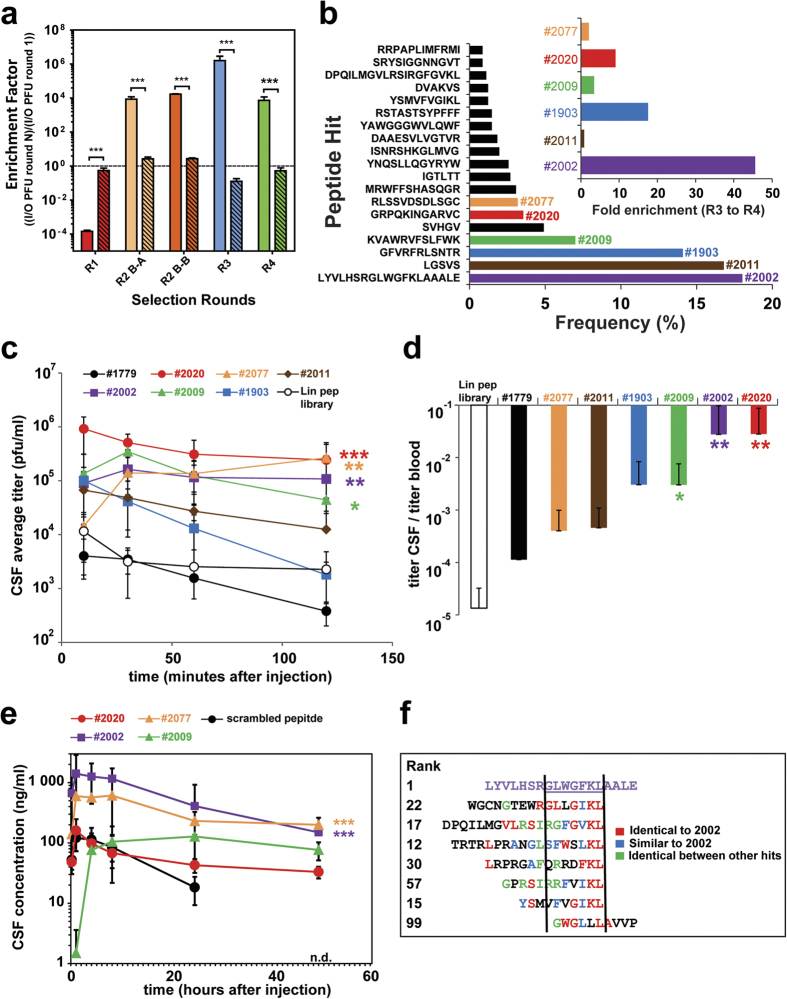
Validation of CSF enriched phage-displayed peptides and CSF transport of biotinylated lead peptides conjugated to streptavidin payload. (**a**) Calculated enrichment factors based on injected (input = I) phage titers (PFU) and determined CSF phage titers (output = O) over all four rounds (R1-R4). The enrichment factors for the last three rounds (R2-R4) were calculated by the comparison with the previous round and the first round (R1) with the wt data. Open bars are CSF and stipple bars are plasma. (***p < 0.001, based on students t-test). (**b**) List of the most enriched phage peptides, ranked based on their relative proportion to all CSF collected phages after the 4^th^ selection round. The six most frequent phage clones are highlighted in colors, assigned with a number and their enrichment factors between the 3^rd^ and 4^th^ selection round (insert). (**c,d**) The six most enriched phage clones of the 4^th^ round, the empty phage and the stock phage peptide library individually analyzed in the CSF sampling model. CSF and blood samples were collected at the indicated time points. (**c**) Equal amounts of 6 candidate phage clones (2 × 10^10^ phages/animal), empty phages (#1779) (2 × 10^10^ phages/animal) and the stock phage peptide library (2 × 10^12^ phages/animal) were tail vein i.v. injected in at least 3 CM cannulated animals each. The CSF pharmacokinetics of each injected phage clone and phage peptide library is displayed over time. (**d**) Display the average CSF/blood ratios of all recovered phage/ml over the sampling time. (**e**) Four synthesized peptide leads and a scrambled control attached through their N-terminal biotin to streptavidin (tetrameric display) and subsequently injected (tail vein i.v., 10 mg streptavidin/kg) in at least three cannulated rats (N = 3). CSF samples were collected at the indicated time points and the streptavidin concentration was measured by an anti-streptavidin ELISA in the CSF (n.d. = not detected). (*p < 0.05, **p < 0.01, ***p < 0.001, based on ANOVA test). (**f**) Comparison of the amino acid sequence of the most enriched phage peptide clone #2002 (purple) with other selected phage peptide clones from the 4^th^ selection round. Identical and similar stretches of amino acids are color coded.

After the third selection round we identified 124 unique peptide sequences from the total 332 CSF-recovered phage clones isolated from both animals (#3.1 & #3.2) (Supplementary Fig. 6a). Sequence LGSVS (18.7%) had the highest relative proportion, followed by wt insert PAGISRELVDKL (8.2%), MRWFFSHASQGR (3%), DVAKVS (3%), TWLFSLG (2.2%) and SARGSWREIVSLS (2.2%). In the final, fourth round we combined the two separate selection branches in three individual animals (Fig. 1c). From the 925 sequenced CSF recovered phage clones of the fourth round we found 64 unique peptide sequences (Supplementary Fig. 6b) in which the relative proportion of wt phages dropped down to 0.8%. The most abundant CSF clones in the fourth round are LYVLHSRGLWGFKLAAALE (18%), LGSVS (17%), GFVRFRLSNTR (14%), KVAWRVFSLFWK (7%), SVHGV (5%), GRPQKINGARVC (3.6%), and RLSSVDSDLSGC (3.2%). The range of lengths of the selected peptides is due to either nucleotide insertion/deletion or a premature stop codon in the library primers when using degenerate codon NNK library design. Premature stop codons generate shorter peptides and selected as they contain favorable aa motifs. The longer peptides are likely a consequence of insertion/deletion in the synthesized library primers. This positions the designed stop codon out of frame and a read-through until a new stop codon appears downstream. Overall, by comparing input with the sampling output we calculated an enrichment factor over all four rounds selection. For the first round of selection we used wt phage titers as unspecific background reference. Interestingly, there was a very strong negative selection of phages in the CSF but not in the blood in the first round (Fig. 3a), possibly because the majority of the library peptide members have either lower probability of passive diffusion into the CSF compartment or a tendency to be withheld or cleared more efficiently from the blood compartment compared to the wt phage. However, in the second round of panning there was a strong selection for phages in CSF in both branches indicating that the previous round enriched for phages that display peptides promoting CSF uptake (Fig. 3a). Again, there was no substantial enrichment in the blood. Also in the third and fourth rounds there was a distinct enrichment of phage clones in CSF. By comparing the relative frequency of each unique peptide sequence between the last two selection rounds we found sequences to be further highly enriched in the fourth selection round (Fig. 3b). A total of 931 tripeptide motifs were extracted from all 64 unique peptide sequences using both directions of the peptides. The most enriched motifs in the fourth round compared to the injected library (cut off: 10% enrichment) were more closely examined for their enrichment profiles over all rounds (Supplementary Fig. 6c). The overall selection pattern shows that the majority of the examined motifs were enriched throughout all previous rounds of both selection branches. Some of the motifs however (e.g. SGL, VSG, LGS GSV) mainly originate from selection branch A whereas others (e.g. FGW, RTN, WGF, NTR) were enriched in selection branch B.

### Validation of single phage clones

From all the enriched phages of the fourth round (Fig. 3b), six candidate clones were selected for further individual analysis in the CSF sampling model. Equal amounts of the six candidate phages, empty phages (no insert) and the stock phage peptide library were injected in three CM cannulated animals each, and the CSF (Fig. 3c) and blood (Supplementary Fig. 7) pharmacokinetics were determined. All phage clones that were examined target the CSF compartment at levels 10–1000 fold higher than the empty control phage (#1779). For example, the CSF titer of clones #2020 and #2077 were about 1000-fold higher than for the control phage. The pharmacokinetic profiles of each selected peptide were all different, yet all possessed a high degree of CSF homing capacity. We observed a constant decline for clones #1903 and #2011 over time whereas clone #2077, #2002 and #2009 increased during the first 10 minutes possibly indicating active transport, but this needs to be validated. Clones #2020, #2002 and #2077 stabilized at a high level whereas the CSF concentration of clone #2009 slowly declined after an initial increase. We next compared the relative occurrence of each candidate in CSF compared to their concentration in the blood (Fig. 3d). Relating the average titers of each candidate in the CSF to their titers in the blood over all sampling time points revealed that three of the 6 candidates are significantly enriched in CSF over the blood. Interestingly, clone #2077 showed increased stability in the blood (Supplementary Fig. 7). To confirm that the peptides themselves are able to actively transport cargo other than a phage particle into the CSF compartment, we synthesized four lead peptides derivatized with biotin at the N-terminus, i.e., where the peptides are linked to the phage particle. The biotinylated peptides (#2002, 2009, 2020 and 2077) were coupled to streptavidin (SA) to generate a multimeric form mimicking to some degree the phage geometry. This format also allowed us to measure the exposure in blood and CSF of SA which serves as a protein cargo for the transport peptides. Importantly, the phage data could in general be replicated when the synthesized peptides were injected in this SA coupled format (Fig. 3e). The scrambled peptide has a much less initial exposure and a faster clearance in CSF with non-detectable levels at 48 hours. To further understand the transport route of these peptide phage clones to access the CSF space, we analyzed the localization of the single phage peptide hits with immunohistochemistry (IHC) detecting the phage particle directly 1 hour after *in vivo* i.v. injection. Notably, clones #2002, #2077 and #2009 could be detected as strong staining in the brain capillaries while the control phage (#1779) and clone #2020 were not detected (Supplementary Fig. 8). This would suggest that these peptides in particular promote brain exposure by crossing the BBB. Additional detailed analysis is needed to verify this hypothesis as the BSCFB route could also be involved. By comparing the aa sequence of the most enriched clone (#2002) with other selected peptides, it is noted that some have similar stretches of aa that could indicate a similar mechanism of transport (Fig. 3f).

### Confirmation studies using a pharmacodynamic readout

**Figure 4:**
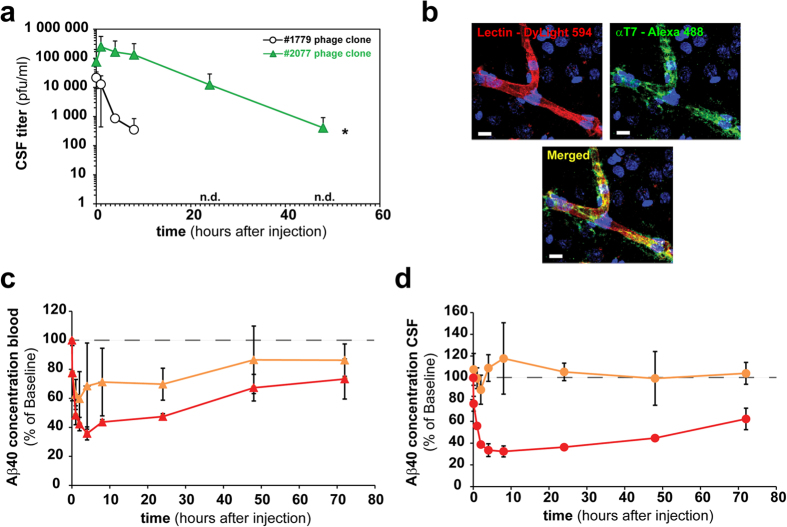
Lead transport peptide enhances brain BACE1 peptide inhibitory activity. (**a**) Long term CSF pharmacokinetic profiles shown for the clonally injected (2 × 10^10^ phages/animal) T7 phage displayed #2077 (RLSSVDSDLSGC) peptide and the insert-less control phage (#1779) in at least three CM cannulated rats each. (**b**) Confocal microscopic image of a representative cortex microvessel in a rat i.v. injected with phage (2 × 10^10^ phages/animal) displaying the #2077 peptide and a vascular counterstaining (lectin). Indicated phage clones were injected into 3 rats and allowed to circulate for 1 hour before perfusion. The brain sectioned and stained with a polyclonal FITC labeled antibody against the T7 phage capsid. 10 minutes before perfusion and subsequent fixation DyLight594 labeled lectin was i.v. injected. Fluorescence images showing lectin (red) stained luminal side of the microvessel and the phage (green) in the capillary lumen and the perivascular brain tissue. Scale bar corresponds to 10 μm. (**c,d**) A biotinylated BACE1 inhibitory peptide alone or in combination with the biotinylated #2077 transport peptide was attached to streptavidin and subsequently i.v. injected (10 mg streptavidin/kg) in at least three CM cannulated rats each. BACE1 peptide inhibitor mediated Aβ40 reduction was measured by an Aβ1-40 ELISA in blood (red) and CSF (orange) at the indicated time points. For better visibility, a dashed line at 100% was drawn in the graphs. (**c**) The percentage blood (red triangles) and CSF (orange triangles) Aβ40 reduction in rats injected with streptavidin attached to the #2077 transport peptide and the BACE1 inhibitor peptide at a 3:1 ratio. (**d**) The percentage blood (red circles) and CSF (orange circles) Aβ40 reduction in rats injected with streptavidin attached with only BACE1 inhibitor peptides. The Aβ concentration for the control was 420 pg/ml (Std. deviation = 101 pg/ml).

Due to its unique plasma profile and substantial increase in CSF over time, clone #2077 displayed on phage was further examined over a longer 48 hour period and could reproduce the rapid increase in CSF and a sustainable level as observed linked to SA (Fig. 4a). As for other identified phage clones, #2077 strongly stained the brain capillaries and when viewed under higher resolution there is a clear co-localization with the capillary marker lectin and potentially some staining within the parenchyma space (Fig. 4b). In order to investigate if a peptide-mediated pharmacological effect in the CNS could be obtained, we performed an experiment where biotinylated version of i) the #2077 transport peptide and ii) a BACE1 peptide inhibitor were mixed with SA in two different ratios. For one combination we used only the BACE1 peptide inhibitor and for the other we used a 1:3 ratio of the BACE1 peptide inhibitor to #2077 peptide. The two samples were intravenously injected and the level of amyloid-β peptide 40 (Abeta40) was determined in the blood and CSF over time. Abeta40 measurement was performed in the CSF as it reflects BACE1 inhibition in the brain parenchyma. As expected, both complexes gave a substantial reduction in Abeta40 levels in the blood (Fig. 4c,d). However, only the sample with the mixture of the #2077 peptide and the BACE1 peptide inhibitor conjugated to SA induced a clear Abeta40 reduction in CSF (Fig. 4c). This data shows that the peptide #2077 is able to transport the 60 KDa SA protein into the CNS and also induce a pharmacological effect by the SA conjugated BACE1 peptide inhibitor.

## Discussion

Phage display has been successfully applied in many areas of biomedical research^17^. This technology has been used for *in vivo* investigations of the vascular diversity^18^,^19^ and in targeting the cerebral vasculature^20^,^21^,^22^,^23^,^24^,^25^,^26^. In this study we have extended the application of this selection approach to the direct identification of not only cerebral vasculature targeting peptides but also to the discovery of candidates possessing active transport properties to cross the brain blood barriers. We now describe the development of an *in vivo* selection procedure in CM cannulated rats and demonstrate its potential for the identification of peptides with CSF homing properties. Applying a T7 phage display 12-mer random peptide library we could show that T7 phages are small enough (diameter ~60 nm)^10^ to be accommodated by the brain vascular barrier for direct crossing of the blood brain barriers or the choroid plexus. We observed that the CSF sampling from CM cannulated rats to be a well-controlled functional *in vivo* screening approach, recovering phages which not only bind to the vasculature but also possess a transport function to pass through the blood-brain fluid barriers. Furthermore, by simultaneous blood collections and applying HTS to both the CSF and blood recovered phages, we confirmed that our CSF selection was not biased by blood enrichment or by amplification fitness between selection rounds. However, the blood compartment is part of the selection procedure as phages that are able to reach the CSF compartment need to survive and circulate long enough in the blood stream to be enriched in the brain. In order to extract reliable sequence information from the raw HTS data we implemented filtering procedures in the analysis workflow that are tailored to platform specific sequencing errors. By incorporating kinetic parameters in the screening approach we confirm the fast pharmacokinetic of wt T7 phages (t½ ∼28 minutes) in blood^24^,^27^,^28^ and also determined their half-life in the CSF (t½ ∼26 minutes). Despite the similar pharmacokinetic profiles in blood and CSF, only 0.001% of the phage blood concentration could be detected in the CSF, indicating low background transmigration of the blood-brain fluid barriers for the wt T7 phages. This work highlights the importance of the first round of selection when using an *in vivo* panning strategy, especially with a phage system that is rapidly cleared from the blood circulation as very few clones are able to reach the CNS compartment. As a consequence, the reduction in library diversity is significantly large in the first round as just a limited number of clones are finally collected in this very stringent CSF model. Multiple selection steps are involved in this *in vivo* panning strategy, such as active accumulation within the CSF compartment, survival of the clones in the blood compartment and the rapid clearance of T7 phage clones within the first 10 minutes from the blood (Fig. 1d & Supplementary Fig. 4M). As a consequence, different phage clones were identified after first round in the CSF, although the same initial library was used in individual animals. This indicates that multiple stringent selection steps with an initial library with high number of library members lead to a strong reduction in diversity. Consequently, stochastic events will be an inherent part of the initial selection process and thus strongly influence the outcome. It is plausible that numerous clones in the initial library have very similar propensities for CSF enrichment. Nevertheless, the selection outcome will be different due to the low number of each specific clone in the initial library even under identical experimental conditions.

The motifs enriched in the CSF were different from those in the blood. Interestingly, we already notice a bias in the first round for glycine-rich peptides in the blood of individual animals. (Fig. 1g, Supplementary Fig. 4e,4f). The phages containing glycine peptides could potentially be more stable and less prone to clearance in circulation. However, these glycine rich peptides were not detected in CSF samples, indicating that the administrated library is subjected to two different selection steps, one in the blood and another allowing for accumulation in CSF. An extensive validation was performed on clones enriched in the CSF resulting from the fourth selection round. It was confirmed that nearly all individually tested clones were enriched in the CSF compared to the empty control phage. We investigated one peptide hit (#2077) in greater detail. It showed prolonged plasma half-life compared to other hits (Fig. 3d & Supplementary Fig. 7) and interestingly this peptide contains a C-terminal cysteine residue. It was recently shown that addition of a cysteine to a peptide may improve its pharmacokinetic properties by binding to albumin^29^. This is currently unknown for peptide #2077 and requires further investigations. Some of the peptides showed a valency dependency in CSF enrichment (data not shown) which is likely related to the display geometry on the surface of the T7 capsid. The T7 system we used displays 5-15 copies of each peptide per phage particle. IHC was performed in the rat cortex on intravenous injected lead candidate phage clones (Supplementary Fig. 8). The data shows that at least three of the clones (#2002, #2009 and #2077) interact with the BBB. If this BBB interaction results in CSF accumulation of these clones or the transport is directly over the BCSFB need to be established. Importantly, we show that selected peptides when synthesized and linked to a protein cargo maintain their CSF transport ability. Conjugation of N-terminal biotinylated peptides to SA essentially replicates the findings obtained with their corresponding phage clones, both in blood and CSF (Fig. 3e). Finally, we show that the peptide lead #2077 was able to promote brain exposure of a biotinylated-BACE1 peptide inhibitor coupled to SA producing a clear pharmacodynamic effect in CNS by significantly lowering CSF levels of Abeta40 (Fig. 4). From a peptide sequence homology search on all hits we were not able to identify any homologs in the databases. It is important to note that the T7 library size was approximately 10^9^ while the theoretical library size for a 12-mer is 4·10^15^. We therefore only sample a small fraction of the diversity space for a 12-mer peptide library which could mean that more optimal peptides could be identified by assessing the nearby sequence space for these identified hits. Hypothetically, one reason that we did not find any natural homologs of these peptides may be explained by deselection during evolution to prevent uncontrolled access of certain peptide motifs into the brain.

Taken together, our results provides a basis for future work to identify and characterize in greater detail transport systems at the brain vascular barriers in their natural environment. The fundamental setup of this approach was based on a functional selection strategy, not only screening for clones with brain vessel binding properties but also involving a key step in which the successful clones possess inherent transport activity through a biological barrier *in vivo* to reach the CNS compartment. The next step is to elucidate the transport mechanism of these peptides and their preferences for binding to regional specific microvasculature of the brain. This could potentially lead to the discovery of novel brain barrier transport pathways and receptors. We anticipate that the identified peptides can either bind directly to brain vessel receptors or to blood circulating ligands that are transported either across the BBB or BCSFB. The peptide vectors with CSF transport activities discovered in this work will be further investigated. Currently, we investigate the brain region specificity of these peptides in order to understand their potential to cross the BBB and/or BCSFB. These novel peptides will be extremely valuable tools toward the potential discover of new receptors or pathways and to engineer novel and efficient brain delivery platforms for large molecules such as biologics.

## Methods

### Surgical implantation of the cannula in the *cisterna magna*

The *cisterna magna* (CM) was cannulated using modification of methods previously described^30^. Anesthetized Wistar rats (200–350 g) were mounted onto a stereotaxic device and a median incision was made on the top of the shaved and aseptically prepared skin of head to expose the skull. Two holes were drilled at the parietal region and mounting screws were secured in the holes. An additional hole was drilled at the external occipital crest and used to stereotactically guide the stainless steel cannula into the CM. Dental cement was applied around the cannula and the screws to hold it in place. After light curing and solidification of the cement, the skin wound was sutured with a 4/0 supramid yarn. Correct placement of the cannula is confirmed by spontaneous flow of cerebrospinal fluid (CSF). The rats were removed from the stereotaxic apparatus, received appropriate post-operative care and analgesic treatment and allowed to recover for at least one week until no sign of blood in the CSF was observed. Wistar rats (Crl:WI/Han) were obtained from Charles River (France). All rats were kept under specific pathogen-free conditions. All animal experiments were approved by the Swiss Veterinary Office Basel-Stadt and were carried out in accordance with the animal permission #2474 (Assessment of active brain transport by measuring the level of therapeutic candidates in the rat CSF and brain).

### Serial collection of CSF and blood from non-anesthetized adult rats

The CM cannulated conscious rats were gently held in the hand. The mandrain was pulled out of the cannula and 10ul of the spontaneous flowing CSF was collected. Only clear CSF samples with no sign of blood contamination or discoloration were included in this study since patency of the cannula was eventually compromised. In parallel ∼10–20 μL blood from a small incision of the tip of the tail was collected in heparin (Sigma-Aldrich) containing tubes. CSF and blood was collected at different time points after intravenous injection of the T7 bacteriophages. Prior to collecting every CSF sample ∼5–10 μL of fluid was discarded, which represents the catheter dead volume.

### T7 phage peptide library engineering

Library was constructed using the T7Select 10-3b vector as outlined in the T7Select System Manual (Novagen, *Rosenberg et al. InNovations 6, 1–6 1996)*. Briefly, random 12-mer insert DNA was synthesized in the following format:

5′-GGGGATCCGAATTCT**(NNK)**_**12**_TAAGCTTGCGGCCGCA-3′ **←**3′-TCGAACGCCGGCGT-5′

NNK codons were used to avoid two stop codons and an over representation of amino acids within the insert. N stands for a hand-mixed equimolar ratio of each nucleotide and K is a hand-mixed equimolar of both adenine and cytosine nucleotides. The single-stranded regions were converted to duplex DNA by continuing incubation at 37 °C with dNTPs (Novagen) and Klenow enzyme (New England Biolabs) in Klenow Buffer (New England Biolabs) for 3 h. After the reaction, double-stranded DNA was recovered by EtOH precipitation. The obtained DNA was digested with EcoRI and HindIII restriction enzymes (both from Roche). The digested and purified (QIAquick, Qiagen) inserts were then ligated (T4 ligase, New England Biolabs) into the predigested T7 vector in-frame after amino acid 348 of the capsid 10B gene. The ligation reaction was incubated at 16 °C for 18 h and subsequently subjected to *in vitro* packaging. The *in vitro* packaging into phages was performed according to the instructions attached to the T7Select 10-3b cloning Kit (Novagen) and the packaging solution was amplified once using E. coli (BLT5615, Novagen) until lysis. The lysate was centrifuged, tittered and frozen as glycerol stocks at −80 °C.

### PCR phage identification

Variable regions of broth or plate amplified phage were directly subject of PCR amplification using in-house designed 454/Roche-amplicon fusion primers. The forward fusion primer contains sequences flanking the variable region (NNK)_12_ (template specific), the GS FLX Titanium adapter A and a four base library key sequence (TCAG) (Supplementary Fig. 1a):

5′-CCATCTCATCCCTGCGTGTCTCCGACTCAGGGAGCTGTCGTATTCCAGTC-3′

The reverse fusion primer also carries a biotin for the attachment on a capture bead and the GS FLX Titanium adapter B necessary for the clonal amplification during the emulsion PCR:

5′-Biotin-CCTATCCCCTGTGTGCCTTGGCAGTCTCAGAACCCCTCAAGACCCGTTTA-3′

The amplicon was then subjected to 454/Roche pyrosequencing according to the 454 GS-FLX Titanium protocol. For the manual Sanger sequencing (Applied Biosystems Hitachi 3730 xl DNA Analyser) the T7 phage DNA was PCR amplified and sequenced with the following primer pairs:

PCR Forward: 5′-AGTACGCAATGGGCCACG-3′

PCR Reverse: 5′-GAGCGCATATAGTTCCTCC-3′

Sequencing Forward: 5′-CAGGAGCTGTCGTATTCC-3′

Sequencing Reverse: 5′-AAAAACCCCTCAAGACCCG-3′

The inserts of individual phage plaques were PCR amplified using the Roche Fast Start DNA Polymerase Kit (according to the manufacturer’s instructions). A hot start (95 °C for 10 min) and 35 amplification cycles at 95 °C for 50 sec, 50 °C for 1 min and 72 °C for 1 min were performed.

### Phage preparation, application, selection and validation

Phages from the library, wt phages, the CSF and blood recovered phages or individual clones were amplified in Escherichia coli BL5615 in either broth TB medium (Sigma Aldrich) or on 500 cm^2^ plates (Thermo Scientific) at 37 °C for 4 h. Phages were extracted from plates by either rinsing the plates with Tris-EDTA buffer solution (Fluka Analytical) or picking the plaques with a sterile pipette tip. Phages were recovered from either the culture supernatant or the extraction buffer by one round of polyethylene glycol precipitation (PEG 8000) (Promega) and resuspended in Tris-EDTA buffer solution.

The amplified phages underwent 2–3 rounds of endotoxin removal using endotoxin removal beads (Miltenyi Biotec) before intravenous (i.v.) injection (500 ul/animal). 2 × 10^12^ phages were injected in the first, 2 × 10^10^ phages were injected in the second and 2 × 10^9^ phages were injected per animal in the third and fourth round of selection. The phage contents in the CSF and blood samples harvested at the indicated time-points were determined by a plaque counting assay according to the manufacturer’s instructions (T7Select System Manual). Phage selection was carried out by i.v. tail vein injection of the purified library or by re-injection of the CSF recovered phages of the previous selection round, and 10 min, 30 min, 60 min, 90 min, 120 min, 180 min respectively 240 min later CSF and blood samples were collected. A total of four rounds of *in vivo* panning were performed in which two selection branches were kept and analyzed separately during the first three selection rounds. All CSF recovered phage inserts of the first two selection rounds were subjected to 454/Roche pyrosequencing whereas all CSF recovered clones of the last two selection rounds were manually sequenced. All blood harvested phages of the first selection round were also 454/Roche pyrosequenced. For the injection of the phage clones the selected phages were amplified in E. coli (BL5615) on 500 cm^2^ plates at 37 °C for 4 hours. The individually picked and manually sequenced clones were amplified in TB medium. After phage extraction, purification and endotoxin removal steps (described above) 2 × 10^10^ phages/animal in 300 μl were i.v. injected into one tail vein.

### Bioinformatic Analysis

Pre-processing of the sequence data and quality filtering: 454/Roche raw data was converted from binary standard flowgram format (sff) to human readable Pearson format (fasta) by using vendor software. Further nucleotide sequence processing was performed with in-house developed C-programs and scripts (unpublished software package) as described in the following. The primary data analysis consisted of stringent multi-step filtering procedures. In order to filter away reads that do not contain valid 12-mer insert DNA sequences, reads were successively aligned to the start tag (GTGATGCTCGGGGATCCGAATTCT), stop tag (TAAGCTTGCGGCCGCACTCGAGTA), and background insert (CCTGCAGGGATATCCCGGGAGCTCGTCGAC) by performing a global Needleman-Wunsch alignment allowing up to 2 mismatches per alignment^31^. Accordingly, reads without start or stop tag and reads containing the background insert, i.e. the alignments that exceeded the allowed number of mismatches, were removed from the library. As for the remaining reads, the N-mer DNA sequence stretches beginning with the start tag and ending before the stop tag are cut out from the original read sequence and processed further (in the following named “inserts”). Upon translation of the insert, the part after the first stop codon from the 5′ prime end was removed from the insert. In addition, nucleotides leading to incomplete codons at the 3′ prime end were also removed. In order to exclude inserts containing only the back ground sequence, translated inserts that start with the amino acid pattern “PAG” were removed as well. Peptides of length shorter than 3 amino acids upon translation were discarded from the library. Finally, redundancies in the insert library were removed and the frequency of each unique insert was determined. The result of this analysis consisted of a list nucleotide sequences (inserts) and its (read) frequencies (Supplementary Fig. 1c and 2).

Grouping N-mer DNA inserts by sequence similarity: In order to overcome 454/Roche specific sequencing errors (e.g. problem of sequencing homopolymer stretches) and remove less trivial redundancies, previously filtered N-mer insert DNA sequences (inserts) were categorized into groups of similar inserts (up to 2 mismatching bases were allowed) by an iterative algorithm defined as follows: Inserts were sorted primarily by their frequency (highest to smallest) and secondary in case of equal frequency by their length (longest to shortest). Consequently, the most frequent and longest insert defined the first “group”. The group frequency was set equal to frequency of the insert. Subsequently, every remaining insert in the sorted list was tried to be added to the group by pair-wise Needleman-Wunsch alignments. If the number of mismatches, insertions, or deletions in the alignment did not exceed the threshold of 2, then the insert was added to the group and the total group frequency was increased by the frequency of added insert. Inserts added to a group were flagged as used and excluded from further processing. If an insert sequence could not be added to an already existing group then the insert was used to create a new group with the corresponding insert frequency and flagged as used accordingly. The iteration finished when every insert sequence was either utilized to form a new group or could be included into an already existing group. After all, grouped inserts composed of nucleotides were finally translated into peptide sequences (peptide library). The result of this analysis was a list of grouped inserts and their corresponding frequencies which accounts for the number of sequenced reads (Supplementary Fig. 2).

Motif generation: On the basis of the list of unique peptides, a library containing all possible amino acid (aa) patterns was generated as follows. Every possible aa pattern of length 3 was extracted from the peptides and together with its reverse pattern added to a universal motif library containing all patterns (tripeptides). The highly redundant motif library was sorted and redundancies were removed. Next, for each tripeptide in the motif library, we verified by applying computational tools its occurrence in the library. If this was the case, then the frequencies of the peptides containing the tripeptide where the motif was found were added and assigned to the motif in the motif library (“motif counts”). The result of the motif generation was a two dimensional array containing all occurring tripeptides (motifs) and their corresponding counts which are a measure for the number of sequencing reads that lead to the corresponding motif upon filtering, grouping and translation of the read as described in detail above.

Normalization of motifs counts and correlation dot-plots: Motif counts were normalized per sample using the following formula


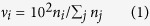


where *n*_*i*_ is the number of reads that contain the motif *i*. Thus, *vi* denotes the frequency in % of reads (or peptides) in a sample that contain motif *i*. P-values of un-normalized motif counts were calculated with Fisher’s exact test. As for the correlation plots of motif counts, Spearman correlations are calculated with R using the normalized motif counts.

In order to visualize amino acid content per position in the peptide libraries, web-logo plots were generated^32^,^33^ (http://weblogo.threeplusone.com). First, the amino acid content per position in the 12-mer peptides was stored in a 20 × 12 matrix. Then, a set of 1000 peptides containing the same relative amount of amino acid per position was generated in fasta sequence format and given as input to the web-logo 3 program which resulted in a graphical representation of the relative amino acid content per position for the given peptide library. For the visualization of multi-dimensional data arrays, heat maps representations were generated by using in-house developed tools in R (biosHeatmap, unpublished R-package). Dendrograms represented in heat maps were calculated by using Ward hierarchial clustering method with Eucledian distance metrics. For statistical analysis of the motif count data, P-values of un-normalized counts were calculated with Fisher’s exact test. P-values for the other data sets were calculated in R with Student’s t-test or ANOVA.

### Immunofluorescent staining of phage

Selected phage clones and insertless phage were i.v. injected via tail vein (2 × 10^10^ phage/animal in 300 μl PBS). 10 minutes before perfusion and subsequent fixation 100 ul DyLight594 labeled lectin (Vector Laboratories Inc., DL-1177) was i.v. injected into the same animals. 60 minutes after phage administration rats were perfused through the heart with 50 ml PBS followed by 50 ml 4% PFA/PBS. Brain samples were additionally fixed by 4% PFA/PBS overnight and immersed in 30% sucrose at 4 °C overnight. Samples were frozen quickly in O.C.T. compound. Frozen samples were subject to immunohistochemistry, performed on 30 um freezing sections blocked with 1% BSA for 2 hours at room temperature and incubated with a polyclonal FITC labeled antibody against the T7 phage (Novus NB 600-376A) overnight at 4 °C. Finally, the sections were then washed 3 times with PBS and observed with a laser confocal microscope (Leica TCS SP5).

### Peptide Synthesis

All peptides with a minimum purity of 98% were synthesized, biotin labeled and lyophilized by GenScript USA. The biotin was coupled via an additional triple Glycine spacer at the N-terminus. All peptides were verified using a mass spectrometer.

### Peptide – Streptavidin – BACE1 coupling and injection

Streptavidin (Sigma S0677) were incubated with a 5x equimolar excess of biotinilated peptides, biotinilated BACE1 inhibitor peptide or a combination (3:1 ratio) of transport peptide and BACE1 inhibitor peptide in 5–10% DMSO/PBS at room temperature for 1 hour prior to injection. 10 mg/kg of the Streptavidin coupled peptides were i.v. injected into one tail vein of cistrna magna cannulated rats.

### Enzyme-linked immunosorbent assays

Concentration of the streptavidin-peptide complex was assessed by ELISA. Nunc Maxisorp microtiterplates (Sigma) were coated with 1.5 ug/ml mouse anti-streptavidin antibody (Thermo, MA1–20011) at 4 °C overnight. After blocking (blocking buffer: 140 nM NaCL, 5 mM EDTA, 0.05% NP40, 0.25% gelatin, 1% BSA) for 2 h at room temperature the plates were washed three times with 0.05% Tween-20/PBS (wash buffer). The CSF and plasma samples were added to the wells diluted in blocking buffer (plasma 1:10000, CSF 1:115). Subsequently the plats were incubated with the detection antibody (1 ug/ml, anti-Streptavidin-HRP, Novus NB120–7239) overnight at 4 °C. After three wash steps, streptavidin was detected by incubation in TMB substrate solution (Roche) for up to 20 minutes. Absorbance was read at 450nm after stopping color development with 1M H_2_SO_4_.

Functionality of the streptavidin-peptide-BACE1 inhibitor complex was assessed by Aβ(1-40) ELISA according to the manufacturer protocol (Wako, 294–64701). Briefly, CSF samples were diluted in standard diluent (1:23) and incubated overnight at 4 °C on 96-well plates coated with capture antibody BNT77. After five wash steps, HRP-conjugated BA27 antibody was added and incubated for 2 hours at 4 °C, followed by five wash steps. Aβ(1–40) was detected by incubation in TMB solution for 30 minutes at room temperature. Absorbance was read out at 450 nm after stopping color development with stop solution. Plasma samples underwent solid phase extraction before Aβ(1–40) ELISA. Plasma was added to 0.2% DEA (Sigma) in 96-well plate and incubated for 30 minutes at room temperature. After washing the SPE plate (Oasis, 186000679) with 100% methanol followed by water, plasma samples were added to the SPE plate and any liquid got removed. The samples were washed (5% methanol followed by 30% methanol) and eluted in 2% NH_4_OH/90% Methanol. After drying the eluates at 55 °C for 99 minutes under constant N_2_-flow, the samples were reconstituted in standard diluent and Aβ(1–40) measured as indicated above.

## Additional Information

**How to cite this article**: Urich, E. *et al.* Cargo Delivery into the Brain by *in vivo* identified Transport Peptides. *Sci. Rep.* **5**, 14104; doi: 10.1038/srep14104 (2015).

## Supplementary Material

Supplementary Information
