# Supplementary material for: Cargo Delivery into the Brain by in vivo identified Transport Peptides
Source: Sci Rep. 2015 Sep 28;5:14104. doi: 10.1038/srep14104 (PMC4585929; doi:10.1038/srep14104)
Supplement: Supplementary Information [file srep14104-s1.pdf]

Supplementary Information – Urich et.al

Cargo Delivery into the Brain by *in vivo* identified Transport Peptides

Eduard Urich[1], Roland Schmucki[2], Nadine Ruderisch[1], Eric Kitas[3], Ulrich Certa[2], Helmut Jacobsen[1], Christophe Schweitzer[1], Alessandra Bergadano[2], Martin Ebeling[2], Hansruedi Loetscher[1] & Per-Ola Freskgård[1]*

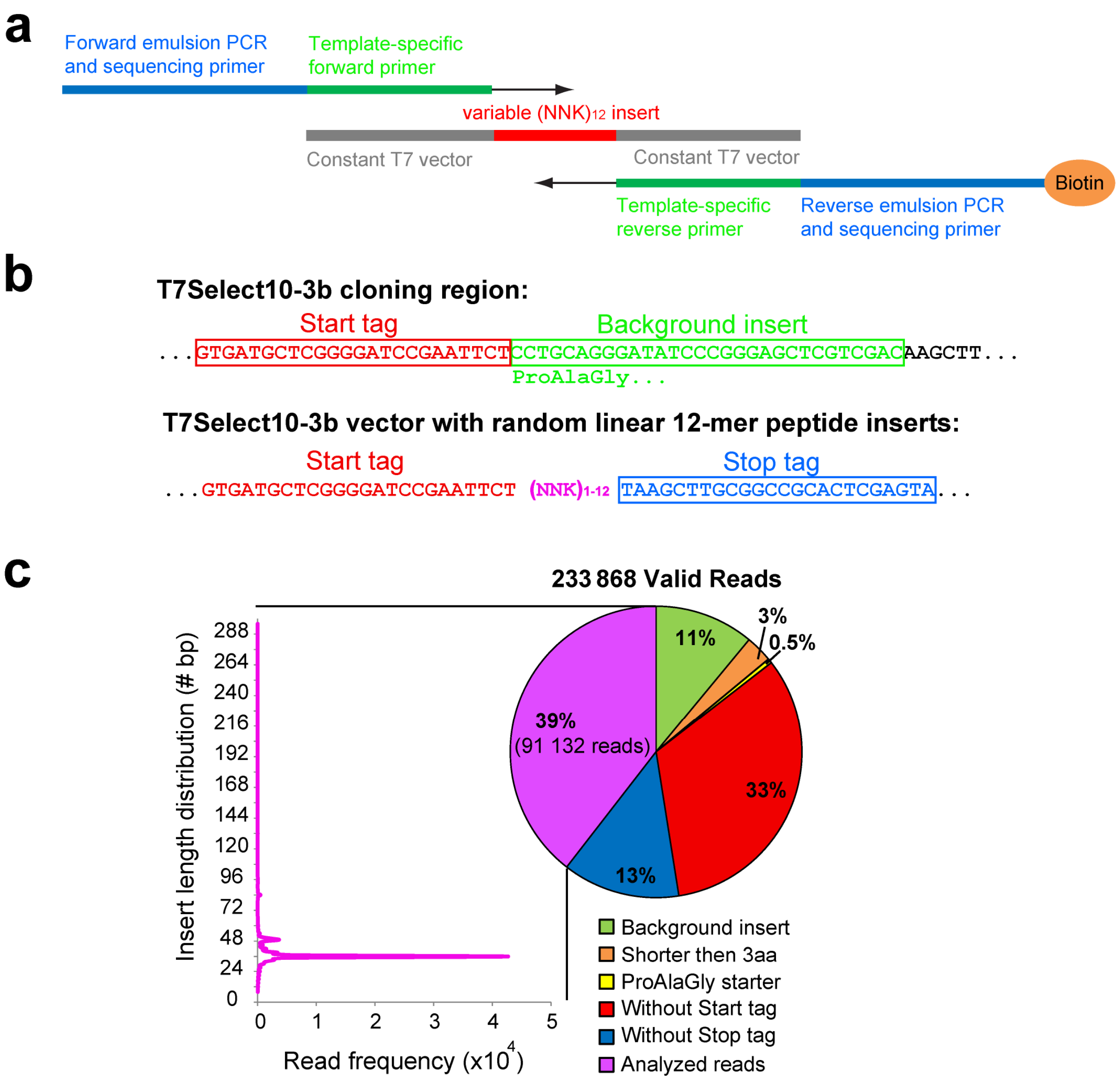

d

	Valid Reads	Reads containing Start Tag	Reads containing Stop Tag	Reads containing Background Insert	Reads that are to short	Inserts starting with "PAG"	Analyzed Reads
Library stock	233868 (100%)	158561 (67.83%)	125677 (53.74%)	26398 (11.29%)	7039 (3.01%)	1108 (0.47%)	91132 (38.97%)
Injected library	120332 (100 %)	72414 (60.18 %)	64186 (53.34 %)	11470 (9.53 %)	3815 (3.17 %)	1320 (1.10 %)	47581 (39.54 %)
1st round animal #1.1 - CSF	89830 (100 %)	36482 (40.61 %)	32645 (36.34 %)	7552 (8.41 %)	1834 (2.04 %)	29 (0.03 %)	23230 (25.86 %)
1st round animal #1.1 - blood	98557 (100 %)	66598 (67.57 %)	61109 (62.00 %)	18789 (19.06 %)	3173 (3.22 %)	1141 (1.16 %)	38006 (38.56 %)
1st round animal #1.2 - CSF	87308 (100 %)	42514 (48.69 %)	37381 (42.82 %)	11497 (13.17 %)	1697 (1.94 %)	691 (0.79 %)	23496 (26.91 %)
1st round animal #1.2 - blood	90936 (100 %)	56904 (62.58 %)	50460 (55.49 %)	7811 (8.59 %)	3278 (3.60 %)	841 (0.92 %)	38530 (42.37 %)
2nd round animal #2.1 - CSF	91600 (100 %)	69049 (75.38 %)	48194 (52.61 %)	6037 (6.59 %)	4778 (5.22 %)	63 (0.07 %)	37316 (40.74 %)
2nd round animal #2.2 - CSF	104336 (100 %)	76317 (73.15 %)	44478 (42.63 %)	7719 (7.40 %)	3953 (3.79 %)	258 (0.25 %)	32548 (31.20 %)
2nd round animal #2.3 - CSF	140263 (100 %)	104408 (74.44 %)	67408 (48.06 %)	11075 (7.90 %)	2335 (1.66 %)	277 (0.20 %)	53721 (38.30 %)
2nd round animal #2.4 - CSF	171767 (100 %)	101395 (59.03 %)	59483 (34.63 %)	8011 (4.66 %)	2119 (1.23 %)	405 (0.24 %)	48948 (28.50 %)

e

	Analyzed Reads<br><br>Inserts Passing the Filter Criteria	Considered Reads<br><br>Inserts with an even Codon Use	Distinct Sequences after Grouping	Distinct 36 Base Pairs long Inserts
Library stock	91132 (100%)	43108 (47.30%)	42580	34193
Injected library	47581 (100 %)	27823 (58.48%)	25411	20583
1st round animal #1.1 - CSF	23230 (100 %)	13362 (57.52%)	1609	964
1st round animal #1.1 - blood	38006 (100 %)	21163 (55.68%)	16458	12346
1st round animal #1.2 - CSF	23496 (100 %)	15384 (65.48%)	764	420
1st round animal #1.2 - blood	38530 (100 %)	23071 (59.89%)	18323	13233
2nd round animal #2.1 - CSF	37316 (100 %)	25106 (67.28%)	924	513
2nd round animal #2.2 - CSF	32548 (100 %)	23040 (70.79%)	797	420
2nd round animal #2.3 - CSF	53721 (100 %)	35076 (65.30%)	678	328
2nd round animal #2.4 - CSF	48948 (100 %)	24286 (49.62%)	780	303

Supplementary Figure 1 – High throughput sequencing fusion-primer engineering and application of the quality filtering steps. (***a***) Schematic representation of the design and alignment of the 454/Roche-amplicon fusion primer used for the PCR amplification and the simultaneously preparation of the variable region for the 454 GS FLX Titanium high throughput sequencing (HTS). The forward fusion primer contains sequences flanking the variable region $(NNK)_{12}$ (template specific) and an adapter sequencing necessary for the initiation of the HTS. The reverse fusion primer in addition carries a biotin for the attachment on capture beads and an adapter sequence which are necessary for the clonal amplification during the emulsion PCR. (***b***) 10-3b vector cloning regions at the end of the T7 capsid gene with the background insert which is replaced by the random peptide library inserts during library generation. Both the 5`- and the 3`- ends of the oligonucleotide strand are flanked by constant regions containing the end of the T7 capsid gene (Start tag) respectively the Stop Codon (Stop tag) terminating the peptide insert transcription. (***c***) Pie chart depicting the proportion of reads coding for the full or parts (ProAlaGly starter) of the background insert ("wt" phage) and reads which are too short or lacking either the Start or the Stop tag. A sample of the stock library was subjected to direct PCR amplification and HTS applying the engineered fusion-primer. A total of 233 868 reads were obtained from the HTS of the stock library of which 39% fulfilled the quality criteria and were further analyzed. The length of those reads were mostly multiples of 3 base pairs (bp) with a peak at 36 bp. (***a & b***) Absolute and relative changes of HTS reads after the quality filtering steps. Prepared samples of the stock library, the injected library (after amplification and purification) and the first two rounds of selection were subjected to HTS. (***a***) All valid reads obtained from HTS underwent 5 subsequent quality filter steps by which short sequences (less than 9 nucleotides) or sequences lacking Start and Stop tag, or containing full or parts (PAG) of the "wt" backbone were discarded. The absolute numbers and the proportions of reads after each filtering step are displayed. (***b***) All reads passing the initial quality filter criteria with even codon use were further analyzed and grouped as described in Material and Methods. Listed are the amounts of all distinct sequences found after grouping and all the 36 nucleotide (12mer) long inserts within the groups.

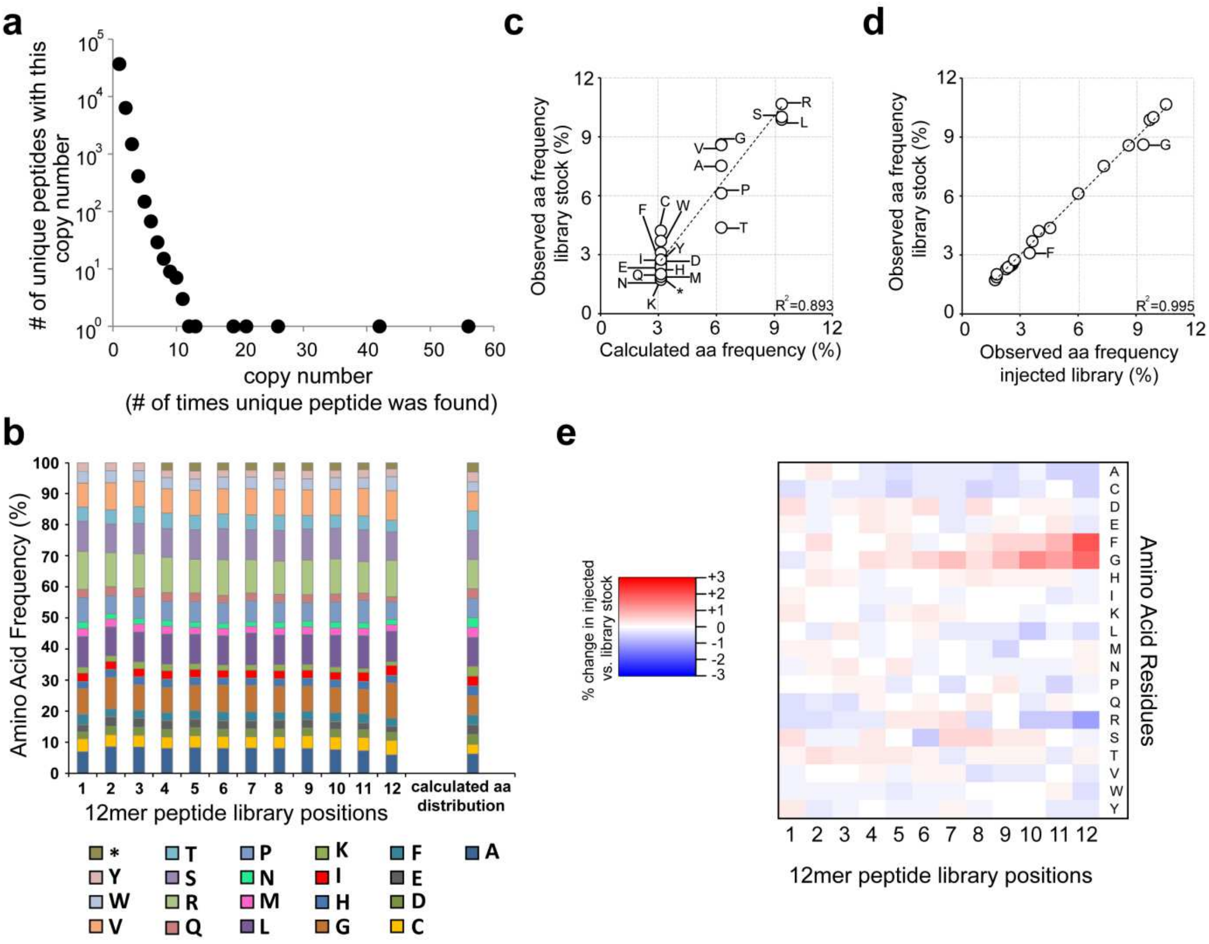

Supplementary Figure 2 – Library diversity conservation despite extensive processing for the *in vivo* application. (***a***) Frequency distribution of all unique sequences within the considered reads of the stock library. The total number of each unique peptide is given along with the number of unique peptides. (***b***) Overview of the relative amino acid composition of all considered 12 amino acid long peptide inserts compared to the theoretical amino acid composition based on the translation of the random $(NKK)_{12}$ inserts. (***c***) The overall amino acid frequencies in the stock library plotted against the theoretical amino acid frequencies as calculated from the number of codons corresponding to each amino acid residue using NNK method of peptide library construction. (***d***) The overall amino acid frequency in the stock library plotted against the averaged amino acid frequency in the injected library. (***e***) Graphic representation of the changes in position-dependent amino acid composition of all considered 12 amino acid long peptide inserts in the stock compared to the injected library.

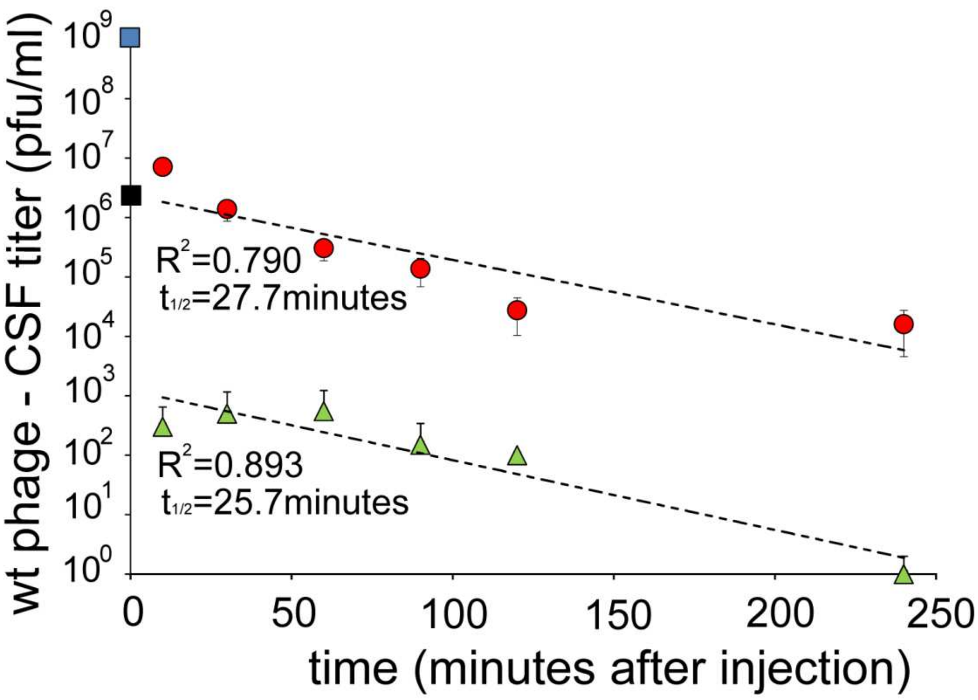

Supplementary Figure 3 – Pharmacokinetic profiles of wt T7 phage in the CSF and blood compartments. Determination of the clearance of wt phage from the blood and their occurrence in the CSF compartment over time. 4 cannulated rats were each i.v. injected with $1x10^{10}$ wt T7 phage. Blood and CSF samples were taken at indicated time points and the average temporal kinetic of phage recovered from blood (red circles) and CSF (green triangles) compartment were extrapolated. The blue square represents the averaged initial blood phage concentrations calculated from the injected phage amount considering the total blood volume based on animal weights. The black square displays the y-axis intercept of the straight line extrapolated from the blood phage concentrations over time.

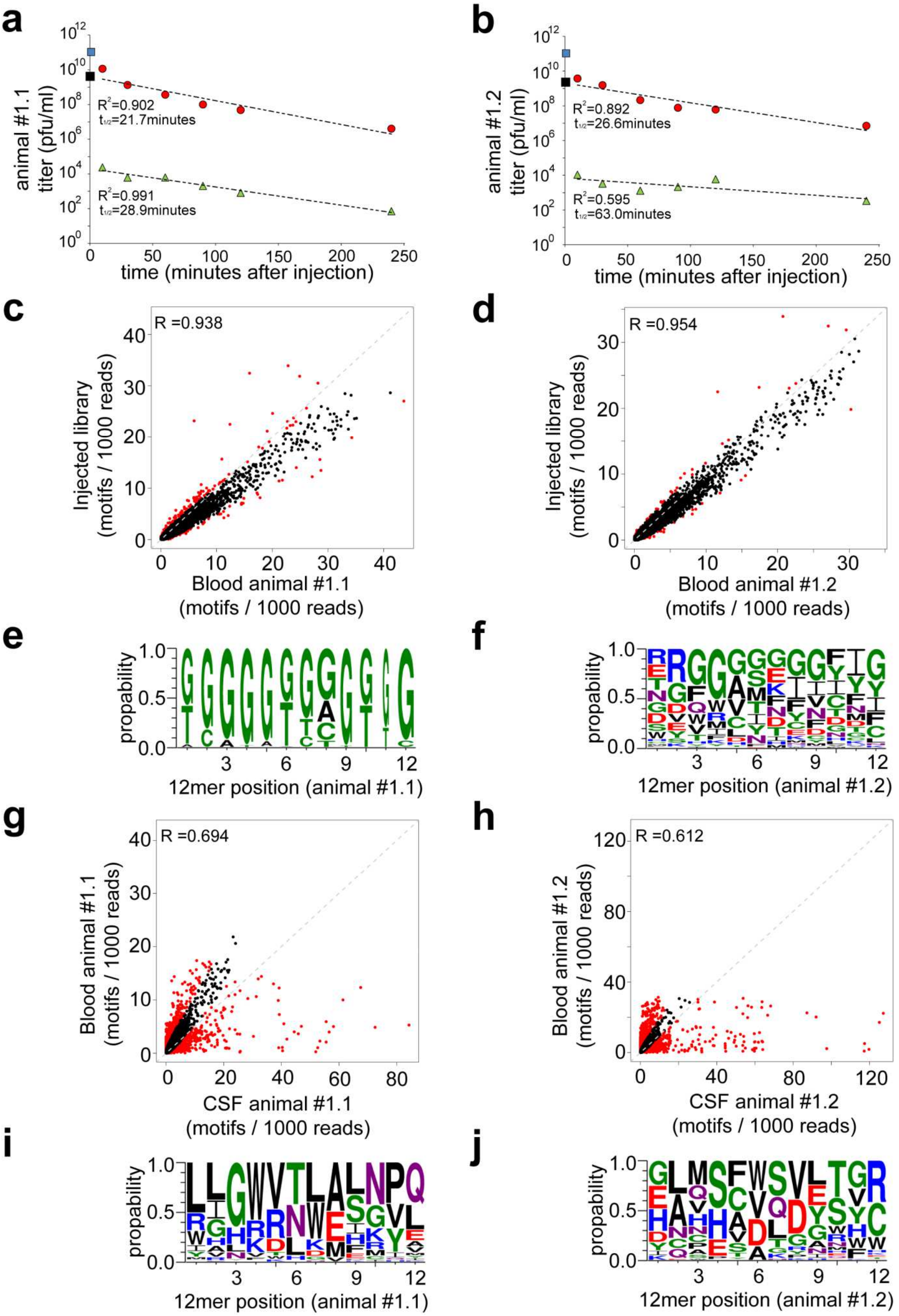

a
animal #1.1
titer (pfu/ml)
R²=0.902
t1/2=21.7minutes
R²=0.991
t1/2=28.9minutes
time (minutes after injection)
b
animal #1.2
titer (pfu/ml)
R²=0.892
t1/2=26.6minutes
R²=0.595
t1/2=63.0minutes
time (minutes after injection)
c
R =0.938
Injected library
(motifs / 1000 reads)
Blood animal #1.1
(motifs / 1000 reads)
d
R =0.954
Injected library
(motifs / 1000 reads)
Blood animal #1.2
(motifs / 1000 reads)
e
propability
12mer position (animal #1.1)
f
propability
12mer position (animal #1.2)
g
R =0.694
Blood animal #1.1
(motifs / 1000 reads)
CSF animal #1.1
(motifs / 1000 reads)
h
R =0.612
Blood animal #1.2
(motifs / 1000 reads)
CSF animal #1.2
(motifs / 1000 reads)
i
propability
12mer position (animal #1.1)
j
propability
12mer position (animal #1.2)

Supplementary Figure 4 – Pharmacokinetic and enrichment profiles after first selection round. The linear T7 phage peptide library was i.v. Injected ($2x10^{12}$ phage/animal) into two independent cannulated rats (animal #1.1 & #1.2). Blood and CSF samples were taken at 10, 30, 60, 90, 120 and 240 minutes after library administration from each animal. (***a & b***) Time course of phage occurrences in blood (red circles) and CSF (green triangles) over time were determined from (***a***) animal #1.1 and (***b***) animal #1.2. The blue square represents the initial blood phage concentrations calculated from the injected phage amount considering the total blood volume based on animal weights. The black square displays the y-axis intercept of the straight line extrapolated from the blood phage concentrations over time. (***c-j***) All CSF and blood derived phage of each animal over time were pooled and high throughput sequenced. (***c,d,g & h***) Relative frequencies and distribution of the motifs representing all possible overlapping tripeptides found within the peptides in both directions. Displayed are the numbers of motifs found within 1000 reads. Motifs which are significantly ($p < 0.001$) enriched in one of the compared libraries are highlighted by red colored dots. (***c & d***) Correlation scatter plots comparing the relative tripeptide motif frequencies of the injected library vs. the blood derived phage of (***a***) animal #1.1 and (***b***) animal #1.2. (***g & h***) Correlation scatter plots comparing the relative tripeptide motif frequencies of the blood and CSF recovered phage of (***i***) animal #1.1 and (***j***) animal #1.2. For better visibility, a dashed diagonal line was drawn in the dot plots. (***e, f, i & j***) Sequence logo representation based on all 12 amino acid long sequences enriched (***e & f***) in the blood over the injected library and (***i & j***) enriched in the CSF over the blood compartment after one round of *in vivo* phage-selection for (***e & i***) animal #1.1 and (***f & j***) animal #1.2. The size of the single letter code represents the frequency of occurrence of that amino acid at a given position. Green = polar, purple = neutral, blue = basic, red = acidic & black = hydrophobic amino acids.

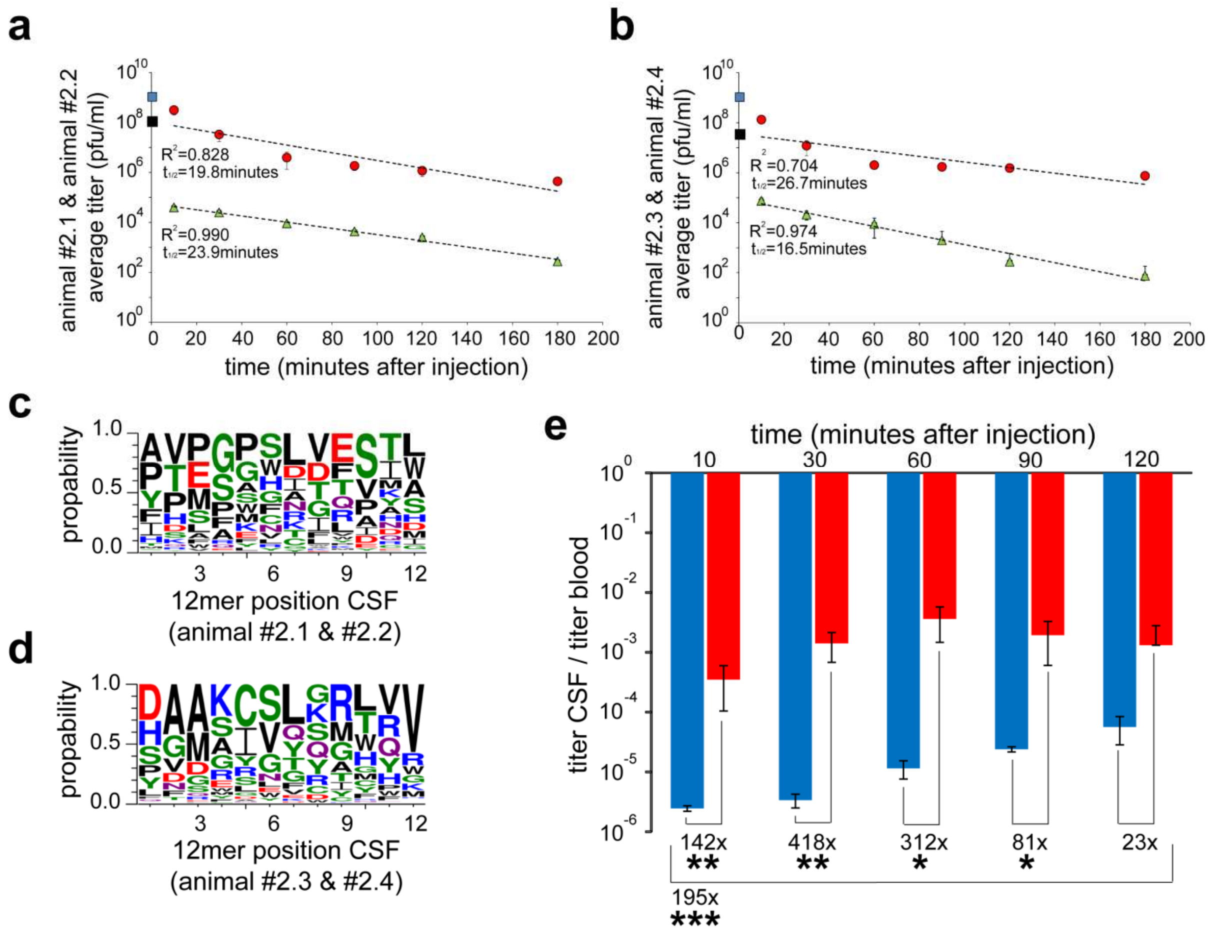

Supplementary Figure 5 – Comparison of the CSF phage frequencies and peptide enrichment between the two first consecutive selection rounds. All CSF recovered phage over time from the 1st round of *in vivo* phage–display selection were pooled, amplified, high throughput sequenced and re-injected together (2x10^{10} phage/animal) into freshly cannulated rats each. Blood and CSF samples were taken and processed at indicated timepoints after i.v. injection. (***a & c***) Animals #2.1 and #2.2 received the CSF recovered phage clones of animal #1.1 whereas (***b & d***) animals #2.3 and #2.4 received the CSF recovered phage clones of animal #1.2. (***a & b***) Averaged time courses of phage occurrences in blood (red circles) and CSF (green triangles) over time were determined from (***a***) animal #2.1 and #2.2 (selection branch A) as well as (***b***) animal #2.3 and animal #2.4 (selection branch B). The blue square represents the initial blood phage concentrations calculated from the injected phage amount considering the total blood volume based on animal weights. The black square displays the y-axis intercept of the straight line extrapolated from the blood phage concentrations over time. (***c & d***) Sequence logo representation based on all 12 amino acid long sequences (***c***) enriched in the CSF of the selection branch A (animal #2.1 and #2.2) over the CSF recovered sequences of animal #1.1 and (***d***) in the selection branch B (#2.3 and #2.4) over the CSF

recovered sequenced of animal #1.2. The size of the single letter code represents the frequency of occurrence of that amino acid at a given position. Green = polar, purple = neutral, blue = basic, red = acidic & black = hydrophobic amino acids. (***e***) The CSF/blood ratio was calculated for each round and sampling time point independently by dividing the averaged recovered phage/ml from the CSF and blood of all animals within a selection round. Displayed are the CSF/blood ratios for the first five sampling time points of the 1st (blue bars) and 2nd round (red bars). The fold enrichment between the two first selection rounds were calculated by comparing the CSF/blood ratios between the two rounds at the given time points as well as for all 5 displayed time points (* p< 0.05, ** p<0.01, *** p<0.001, based on students t-test of log2).

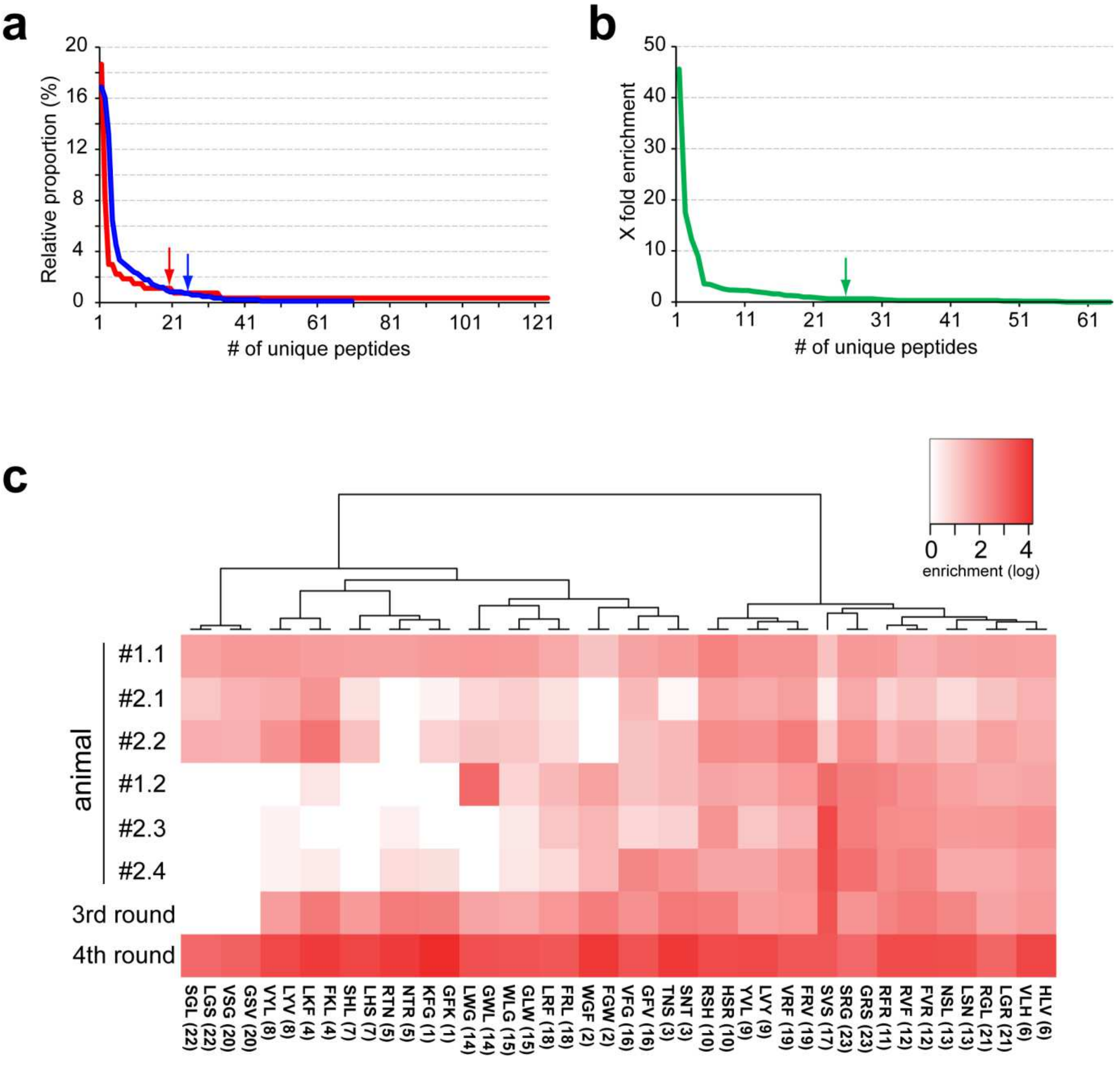

Supplementary Figure 6 – Relative frequency distribution of unique peptides between the last two selection rounds and the motif enrichment pattern during all selection rounds. (*a*) The relative proportion of unique peptides related to all sequenced clones of the 3rd (red line) and 4th round (blue line). The positions of the insert-less (empty) phage are displayed by an arrow. **(*b*)** Displayed is the fold enrichment of all unique sequences identified in the 4th round compared to their relative proportions in the 3rd round. The enrichment of the insert-less phage is highlighted by a green arrow. **(*c*)** Enrichment profiles of the most enriched motifs (compared to the injected library) in the 3rd and 4th round as well as the six animals from previous selection rounds. The course of enrichment is displayed as a head map in which the degree of enrichment correlates with the color intensity. The bracketed numbers behind the tripeptide motifs are the frequency of occurrence in the fourth round. The clustering of the enriched motifs originating from distinct peptide sequences is visualized by a phylogenetic tree.

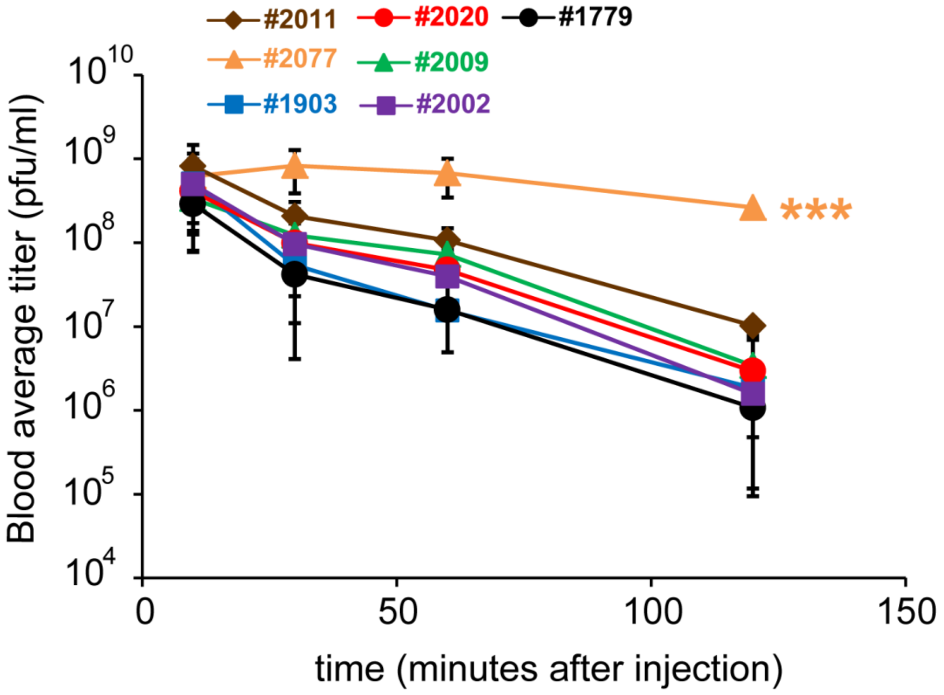

Supplementary Figure 7 – Pharmacokinetic profiles of the CSF enriched phage clones in the blood. Equal amounts of 6 candidate phage clones (2x10^{10} phage/animal) and the insert-less (empty) control phage (#1779) (2x10^{10} phage/animal) were teil vein i.v. injected in at least 3 CM cannulated rats each. Blood samples were collected at the indicated time points. The average temporal kinetics of phages recovered from the blood compartment over time are displayed (*** p<0.001, based on ANOVA test).

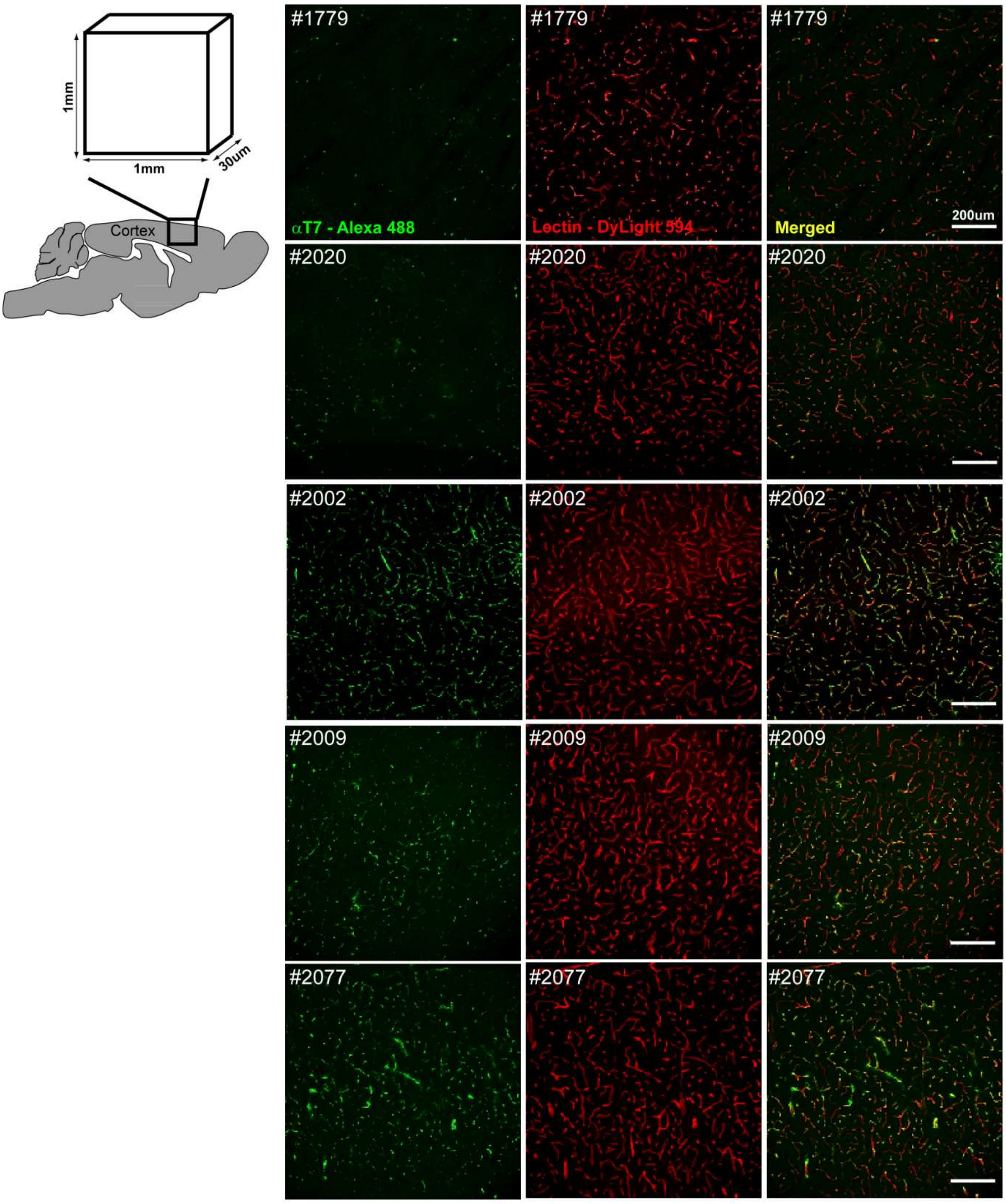

Supplementary Figure 8 – Brain microvessel association of a phage synthesizing a peptide with high occurrence in CSF. Representative confocal microscopic images of the cortex microvessels in a rat teil vein i.v. injected with phage clones ($2x10^{10}$ phage/animal) displaying the CSF enriched peptides or the insert-less control phage (#1779) ($2x10^{10}$ phage/animal). Indicated phage clones were injected into 3 rats and allowed to circulate for 1 hour before the brains were withdrawn and

stained with a polyclonal FITC labeled antibody against the T7 phage capsid. 10 minutes before perfusion and subsequent fixation DyLight594 labeled lectin was teil vein i.v. injected. Fluorescence images were recorded showing lectin (red) stained microvessels and the associated phage (green). Displayed pictures are emerging from six z-stacks taken in a distance of 5um. Scale bar corresponds to 200um.
